# Comparative perspectives on wrongful convictions: Insights for innocence research and reforms in the USA, England and Wales, and the Netherlands

**DOI:** 10.1016/j.fsisyn.2026.100718

**Published:** 2026-08-11

**Authors:** Michael P. Toglia, Rebecca K. Helm, Garrett L. Berman, Shelley L. Thibodeau, Annelies Vredeveldt, Jennifer M. Schell-Leugers

**Affiliations:** a Cornell University, USA; b University of Exeter, England; c Roger Williams University, USA; d Conviction Integrity Review, Fourth Judicial Circuit, State Attorney's Office, Jacksonville, FL, USA; e Vrije Universiteit Amsterdam, Netherlands; f Maastricht University, Netherlands

**Keywords:** Wrongful conviction, Exoneration, Innocence research, Criminal justice systems, Conviction integrity unit

## Abstract

To illustrate the prevalence of wrongful convictions and their causes, we report critical analyses of international archival databases documenting known exonerations in the USA, England and Wales, and the Netherlands. We suggest that unjust convictions should be viewed as system failures in both adversarial and inquisitorial systems. A common theme that weaves throughout the article is that there are (often procedural) differences across the jurisdictions, yet there are many similarities in recognised wrongful conviction cases. Perhaps most striking is that not only are wrongful convictions a global problem, but that many wrongful convictions share common features regardless of the underlying jurisdiction. We identify these features, which include, for example, false confession, mistaken eyewitness identification and official misconduct, and which frequently co-exist. We also examine the relationship between other variables of interest (e.g., race, crime type, DNA) and wrongful convictions in each jurisdiction. The comparative approach allows for varying jurisdictions to learn from each other in order to ultimately enhance the efforts of researchers, policy makers, and criminal justice practitioners in transforming their systems. The transformations should in large measure target formulating future policies and reforms. Aside from the differences, the similarities across databases further indicate that these findings are robust and provide an important first step in comparing databases across varying jurisdictions and developing common reforms.

## 1 Overview, purpose and framing

The focus of this special issue, wrongful convictions, represents one of the most far-reaching failures of modern criminal justice systems [1]. In this article, we focus specifically on wrongful convictions as they pertain to the conviction of people who by virtue of exoneration are now recognised to be (or to be very likely to be) innocent. Briefly expanding on this statement's three concepts is necessary as [2] have warned, that literature on such convictions frequently uses relevant terms interchangeably and/or fails to even define them. One of the terms in question is “exoneration”. It will become clear that the three registries we discuss vary somewhat in how it is defined, though all refer to overturning a wrongful conviction, our focus as just noted. Broadly, a wrongful conviction is when an innocent person is found guilty of a crime that she/he did not commit or actually did not occur. Finally, we note that relief from a wrongful conviction does not automatically mean a defendant is deemed innocent. They may be "presumed" innocent as opposed to proven as actually innocent, Actual innocence is a “legal or factual determination that the person did not commit the crime.” [3]. We have sought to use these three terms carefully in accordance with the definitions above, while explicitly providing inclusion criteria for each registry.

Continuing with the focus on the serious consequences of wrongful convictions, the recognised conviction of innocent people undermines public confidence in legal institutions and the legitimacy of adjudicative procedures. Those who are wrongly convicted frequently suffer extreme harm, which persists following their convictions being overturned (see e.g., Ref. [[4], [5], [6]]), and secondary victims (including the families of the wrongfully convicted) both pre- and post-exoneration, also often suffer significant and enduring harm (e.g., Ref. [7]). Of course, when innocent people are convicted in cases involving crimes that they did not commit, the actual perpetrators remain free, possibly to commit further crimes. Thus, wrongful convictions can compromise public safety as well as imposing unjust treatment of the innocent.

Over the past four decades, the systematic study of wrongful convictions has increased significantly (www.innocenceproject.org; [8]). Studying the causes of wrongful convictions is not new. Nearly a hundred years ago, Borchard [9] published his ground-breaking book, *Convicting the Innocent.* He was, to our knowledge, the first researcher to systematically document wrongful convictions and leverage his findings to promote the necessity for legal reforms. Borchard collected 65 documented cases in the USA wherein judicial error led to the wrongful conviction of an innocent defendant charged with a serious crime. Among the several causes of these errant convictions were false confession and perjury. However, Borchard noted that mistaken identification was the primary contributing factor, occurring in 45% of the cases he examined. His cataloguing of errors was significant as a first step towards analyzing known instances of wrongful convictions. This in turn provided some evidence to discredit the dominant belief in his time that such convictions were rare; and therefore, served as an early appeal to legal scholars to reframe their inquiries concerning innocence. Borchard urged that it was important not only to ask whether innocent people are wrongfully convicted but also to ask why innocent people are found guilty, and to explore how the criminal justice system should be reformed to reduce these mistakes.

In the decades following this work, other US scholars scrutinized and documented relatively small sets of cases of injustice to provide insight into causes underlying wrongful conviction, all noting the prominence of mistaken witness identification, a discovery that is not new, as we shall see (e.g., Ref. [[10], [11], [12], [13]]). However, the most significant progress in the systematic examination of wrongful convictions has occurred relatively recently with the creation of databases logging, categorizing, and analyzing the characteristics of known wrongful convictions.While these databases include a set of convictions that have been overturned by appeals courts based on the standards those courts apply and not necessarily on a clear “innocence” standard, they typically include cases in which, as a result of inclusion criteria and the stringency of appeals courts standards, tend to involve strong indicators of innocence (see, for e.g., Ref. [14] in the England & Wales context). Momentarily, several prominent exoneration databases will be noted. An important cautionary point is that registry data should not be conflated with the prevalence of wrongful convictions. Trusted research [15] suggests that the occurrence of unjust convictions in capital cases, that included serious felonies, is about 1–5%, with a few well-designed studies estimating rates at about 11% in specific contexts, such as wrongful conviction cases in which DNA evidence was introduced. The variability is wide because estimates depend on the methods and case types studied. For a detailed treatment of this frequency issue, we recommend readers consult [16]. Regardless of the actual frequency with which incorrect convictions occur, we concur with the belief that wrongful convictions are very likely undercounted. What remains constant, however, is that wrongful prosecutions, verdicts and imprisonments represent serious global problems that require our utmost attention; the characterization that these grave concerns are worldwide will be substantiated momentarily.

In 2012, the National Registry of Exonerations (NRE), a project of the University of Michigan Law School, Michigan State University College of Law, and the University of California Irvine Newkirk Center for Science & Society, was established to collect, analyze, and disseminate information about *all* known exonerations of wrongfully convicted, innocent defendants in the United States. This was, to our knowledge, the first attempt to comprehensively log and categorize wrongful convictions in a region (although prior to this the Innocent Project maintained a database of DNA exonerations only across the United States, something which it continued to do until 2020, then shifted to tracking “IP Successes” that include both DNA and non-DNA cases). The NRE database contains 4272 cases, split into 475 pre-1989 cases and 3797 post-1989 cases when DNA testing became available [17], and has been instrumental in assisting legal practitioners, researchers, and policymakers in their attempts to understand and remedy the flaws in the criminal justice system that lead to innocent people being convicted of crimes. Recently, databases documenting wrongful convictions in other regions have been created. These include the United Kingdom Miscarriages of Justice Registry (MJR; founded in 2021 and documenting recognised wrongful convictions [typically referred to as miscarriages of justice in the UK system] in the three UK jurisdictions – England and Wales, Scotland, and Northern Ireland), the Canadian Wrongful Convictions Registry (founded in 2023 and documenting recognised wrongful convictions in Canada) and the European Registry of Exonerations (EUREX; founded in 2024 and documenting recognised wrongful convictions across twenty European jurisdictions).

Some inroads, including data, have been made to examine erroneous convictions in world regions besides Europe and North America as well. For example, Espag and Tredoux [18] recently documented erroneous convictions in South Africa. They employed an aggregated case study design wherein they systematically reviewed, collected and qualitatively analysed a small set of such convictions. The authors specifically focused on cases involving eyewitness misidentification and/or false confessions, both of which are common causes recorded in all global registries with which we are familiar. Though a registry has yet to be established, Roman and Duce (2024) reviewed 50 scholarly papers addressing miscarriages of justice, including wrongful convictions, between 2010 and 2023 in Latin America. The top contributing factors extracted in the qualitative analyses were eyewitness misidentification, inadequate legal representation, misconduct and false confession. Similarly, a systematic review of wrongful convictions in Asian countries was conducted by [19]. They located 36 English peer-reviewed articles published between 2010 and 2021 that were examined to shed light on the extent and response of Asian countries to wrongful convictions and determining the primary causes of those wrongful convictions. Nearly all publications focused on China and Japan, because a paucity of studies reviewed other Asian countries. These analyses isolated false confessions, torture, misconduct, and eyewitness identification errors as the top factors contributing to wrongful convictions.

In the present paper, we provide an in-depth comparison of three jurisdictions: the USA, England-Wales and the Netherlands. Focusing on a limited number of jurisdictions enabled a more detailed analysis than would have been possible with a broader set of less comparable countries. We selected these jurisdictions because they represent a range of legal traditions (from adversarial to inquisitorial), acknowledge wrongful convictions as a systemic problem, have mechanisms for evaluating plausible wrongful convictions, have established reforms, and demonstrate a willingness to utilize forensic-science-informed review processes. The cases contained in these databases have been analysed to provide insight into the factors associated with wrongful convictions in each region (see e.g., Ref. [14,[20], [21], [22]]). However, to date, there has been relatively little utilization of the set of complementary databases in comparative analyses (although see Ref. [23]). Such analyses have the potential to be valuable, indeed essential, in achieving both a better understanding of the causes of wrongful conviction and how they operate in different contexts, and in learning from different approaches adopted by varying jurisdictions in designing and implementing reform.

We draw on registry data, and broader insights, to examine wrongful convictions from a comparative, global perspective. We begin by introducing the criminal justice systems in each of the three jurisdictions, demonstrating some key differences in procedure relevant to criminal convictions and appeals. We selected these jurisdictions because they allowed us to draw upon these registries documenting erroneous conviction cases in each jurisdiction to identify (1) basic descriptive information on wrongful convictions (including, where available, relating to gender, offence, race/ethnicity, and use of DNA), (2) key causes that have been associated with wrongful convictions in each region and their relative prominence, and (3) some additional (non-comprehensive) findings that we refer to as exploratory analyses that provide insight into the body of wrongful convictions cases in specific registries. Following the descriptors, causes and findings across the three registries, we briefly identify disparities between jurisdictions but note the challenges in interpreting these differences and the need for future work. We also identify striking similarities between jurisdictions, despite their differing legal systems. We draw on these similarities to suggest that jurisdictions can learn from reforms elsewhere and discuss how these parallels provide an important blueprint for new and developing databases (such as Africa, Latin America and Asia).

This section is followed by a brief review of reforms in each jurisdiction that may be drawn on more broadly in efforts to address wrongful convictions.

## 2 Introducing the systems: the USA, England and Wales, and the Netherlands

The criminal justice systems in different jurisdictions vary not only in relation to geography and population, but also key aspects of criminal investigation, trial, and appeals, that are important in contextualizing data on wrongful conviction. Below we provide a brief introduction to each system and its most important elements in relation to wrongful convictions. However, it is important to note that the differences between jurisdictions are expectedly numerous, and a full discussion of underlying systems and their differences is beyond the scope of this work.

### 2.1 The USA

The criminal justice system in the United States is an adversarial system, guided by the Constitution of the United States, the individual state constitutions that may afford additional safeguards, federal and state statutes (laws), and court rulings that interpret the meaning and applicability of the Constitution and federal and state statutes. There are more than 18,000 independent law enforcement agencies across the country that are responsible for investigating crimes committed within their individual jurisdictions. Law enforcement agencies are bound by Constitutional protections, such as a defendant's right against self-incrimination, protection from unlawful search and seizure, and the right to assistance of legal counsel. Once a criminal investigation has begun, the law enforcement agency controls the scope of the investigation and the mechanisms used to collect evidence. An arrest triggers review by the prosecutor's office. If appropriate, the prosecutor brings the charges based upon the evidence collected. Likewise, defendants are afforded protection during the pretrial and trial process, such as the right to a bail hearing, the right to assistance of counsel, and the right to confront witnesses. The trial judge is the arbitrator of what evidence is admissible, and it is common for defendants to test the sufficiency of the evidence by filing a motion to dismiss the charges, or by filing pretrial motions to suppress evidence or information gathered during the investigation phase for being unlawfully obtained, irrelevant, or more prejudicial than probative.

Continuing along these lines, the USA's system of justice is historically rooted in English common law traditions. The USA's adversarial model developed in contrast to the more judge-centered inquisitorial systems of continental Europe (for an introduction to basic concepts and institutions in America's system, see Ref. [24]). In cases involving offenses punishable by 6 months or more in jail, defendants have a constitutional right to trial by jury under the Sixth Amendment of the United States Constitution. Procedures surrounding trial by jury differ by state – juries often contain 12 jurors but may operate with fewer (see Ref. [25]), and decisions must be unanimous [26]. Procedures for selecting jurors can be complex and vary by state, with most states allowing “peremptory challenges,” through which they can eliminate potential jurors without giving a reason (for some comparative discussion, see [27]). At trial a range of evidential rules govern the introduction of evidence (see Ref. [28], in the federal context), witnesses can be questioned through direct examination, cross-examination, and (sometimes) re-examination, and there is a presumption of innocence using the highest standard of proof (prosecutors must prove defendants guilty beyond a reasonable doubt). Defendants face strong incentives to plead guilty in exchange for a reduced sentence (or lesser charges), a process known as ‘plea bargaining’. This method successfully resolves more than 90% of criminal cases across the USA (for a discussion of the plea system, see Ref. [8]; on incentives to plead, see also [29]). Where a plea agreement was struck or a case went to trial, an appeal is possible, as described next.

Once convicted after trial and under limited circumstances after a plea (e.g., see Ref. [30]) defendants are entitled to a direct appeal to challenge the rulings of the trial court. In the USA, appellate courts look only to the record created at the lower court level for adherence to the law, evaluate procedural adherence rather than factual accuracy, and generally will not reweigh the credibility of a witness or their testimony. This appellate structure places a high level of deference on the findings of juries and the rulings of the trial judges. In the USA, once a jury returns a guilty verdict, the legal system shifts from a presumption of innocence to a presumption of guilt, making it difficult to overturn convictions on factual grounds. When the first 200 DNA exonerations in the USA were reviewed, only 14% (28 individuals) received any form of relief from the appellate courts. Appellate relief generally entails reversal back to the trial court for additional proceedings or retrial based upon procedural and constitutional errors, and not on factual innocence grounds. The remaining 86% of cases reviewed were affirmed by the appellate courts, often with the court citing “overwhelming” evidence of guilt, or errors that were “harmless” because they were unlikely to impact the verdict [31,32]. In sum, the appellate process is designed to ensure the rules were followed rather than ensure the conviction was factually correct.

### 2.2 England and wales

The criminal justice system in England and Wales is also adversarial (for an introduction to basic concepts and institutions in the system, see Ref. [33]). A range of rules and recommendations govern police investigation and the gathering of evidence, most notably the Police and Criminal Evidence Act 1984, which contains detailed codes of practice relating to, among other things, protocols in police interviews and identification procedures.

Cases involving less serious offending (“summary only” offenses and some “either way” offenses) are heard by a judge or a panel of lay magistrates in the magistrates' court (for more information on the magistrates’ court, see Ref. [34]). Cases involving more serious offending are heard by a jury, presided over by a judge, in the Crown Court. The jury system in England and Wales differs in important respects from the jury system in the United States - jurors in England and Wales can only be challenged for cause, jurors can reach a verdict either unanimously or by a “super-majority” (at least 10-2), and judges have a more active role at trial, including through summarizing evidence (for a detailed discussion of differences, see Ref. [35], Chapter 2). As in the United States, a range of evidential rules govern the introduction of evidence at trial (for a summary, see Ref. [36]), witnesses can be questioned through direct examination, cross-examination, and (sometimes) re-examination, and there is a presumption of innocence and high burden of proof (people must be proven to be guilty beyond a reasonable doubt). Defendants are incentivized to plead guilty through sentence discounts awarded by a judge (and sometimes through charges being dropped by a prosecutor), although incentives are more modest than in the USA, and around 70%-80% of defendants plead guilty (see Ref. [37]).

The avenues through which a person can appeal their conviction depend on the court in which they were convicted and whether they pleaded guilty (for a full review of current appeals procedure, see Ref. [38]). There is no ground of appeal based on innocence (for discussion, see Ref. [39]) and appealing a conviction following a guilty plea is extremely difficult (e.g., Ref. [40]). Cases are either re-heard on appeal (where a conviction occurred in the magistrates' court) or assessed based on a ‘safety’ standard in the Court of Appeal. A conviction can be overturned if it is found to be ‘unsafe’ on the basis of either legal or procedural irregularity or factual doubts about guilt (with ‘miscarriages of justice’ often being confined to the latter). In addition to the traditional avenues to appeal, all defendants (regardless of plea, and usually once they have exhausted other avenues for appeal) can also apply to the Criminal Cases Review Commission (CCRC), who have the power to refer a conviction back to appeals courts (either the Crown Court or, more commonly, the Court of Appeal) where there is a ‘real possibility’ that the appeals court will overturn the conviction (Criminal Appeals Act 1995). Once the CCRC has referred a case, it is considered by the relevant appeals court in line with normal appeals procedure.

### 2.3 The Netherlands

The Dutch legal system is rooted in an inquisitorial tradition in which judges have both investigative and adjudicative responsibilities (for comprehensive reviews, see Ref. [[41], [42], [43]]). Rather than facilitating an adversarial contest between prosecution and defense, the Dutch criminal procedure centers around fact-finding. Judges rely heavily on a prosecutor-led process wherein a comprehensive case dossier is compiled during the investigation, which contains police procedures, witness statements, expert reports, and other evidence. Because this dossier provides an extensive factual basis, Dutch judges rarely question witnesses or experts during the hearing. Although the prosecutor and defense counsel present their positions, these are often less polarized than in accusatorial systems, reflecting a broader emphasis on compromise. The judiciary, acting without a jury, is ultimately responsible for reconstructing events and determining whether the defendant's guilt is convincingly established and what sentence should be imposed. There is currently no established ‘guilty plea’ system in the Netherlands.

In the Netherlands, cases that are expected to involve a prison sentence of more than one year or are considered particularly complex or socially significant, are always heard by a panel of three judges (as opposed to a single judge). Further, a judgement by a Court of First Instance (*Rechtbank*) can always be appealed by either the prosecution or the defense or both, in which case it will go to a Court of Appeal (*Gerechtshof*) with three new judges who conduct a completely new review of the case. Their judgment can be further appealed for review by the Supreme Court of the Netherlands (*Hoge Raad)*, but this court only considers matters of law (e.g., whether a legal rule was applied appropriately), as opposed to the facts of the case. Closed cases can be reopened, but this rarely happens because the requirements are very strict: new evidence that was not available to the judges at the time of the appeal judgment (a *novum*). This may involve newly discovered DNA, new techniques to analyze DNA, new witnesses coming forward, or occasionally experts providing new insights into crucial elements of a case. Decisions as to whether to reopen a case are made by the Supreme Court. An independent advisory committee, the Advisory Committee on Closed Criminal Cases (ACAS) exists to provide advice about when additional fact-finding should be undertaken to review potential wrongful convictions. However, unlike the CCRC, the ACAS does not have the power to reopen a case. Table 1 summarizes differences and similarities in the three systems.

**Table 1:** Comparative overview of criminal justice systems and procedures.

Feature	United States	England and Wales	Netherlands
**System orientation**	Adversarial	Adversarial	Inquisitorial
**Charging authority**	Prosecutor; sometimes grand jury indictment depending on jurisdiction	Prosecuted by the Crown Prosecution Service after police investigation	Prosecuted by the Public Prosecution Service (Openbaar Ministerie)
**Primary trial format**	Jury trial for cases involving a sentence over 6 months	Jury trial in Crown Court for more serious offending. Trial in magistrates' court for less serious offending.	Bench trials before professional judges
**Decision maker**	Jury (typically 12 citizens, but can be as low as 6)	Jury (12 citizens)	Professional judge(s); no jury
**Verdict rule**	Unanimous verdict required	Majority verdict allowed (typically 10–2 or 11–1 after deliberation)	Majority decision among judges
**Jury selection procedures**	Extensive voir dire with attorney questioning and challenges	Limited questioning; challenges generally restricted to cause	Not applicable (no jury system)
**Typical judicial role**	Neutral arbiter of procedure and law	Neutral arbiter of procedure and law, but responsible for summing up which can include comment on the weight of evidence	Judges play a more active evaluative role in evidence

## 3 Descriptive analyses and causes of wrongful convictions

### 3.1 The USA

The NRE's main database covers the post-1989 period (the period following the first DNA exoneration) and, as noted earlier, at the time of writing, includes 3797 cases in which an innocent person has been exonerated (i.e., convicted of a crime but later relieved of all legal consequences of that conviction through a decision by a prosecutor, a governor or a court, after new evidence of his or her likely innocence was discovered; for full inclusion criteria and information on registry compilation, (see Ref. [44]).

#### 3.1.1 Descriptive information

Regarding sex, the NRE is heavily slanted towards males, numbering 3458 (86.6%), with females only accounting for 329 (13.4%) cases. In terms of race data, it is clearly skewed in the direction of Black individuals (53.6%). White exonerees were next at 31.1%, followed by Hispanic (12.9%), Asian (.009%). Native American (.007%) and Other/Don't Know (.007%). Noting only major crimes, these offenses include murder (36.7%), sexual assault (10.1%), child sex abuse (9.1%), and attempted murder (2.7%). The average time spent incarcerated across all exonerees was substantial (*M* = 9.35; *SD* = 9.74) with a combined total of 35,457 years. When examining time served by race, the average time unfairly spent in prison for Black exonerees was over fourteen years (*M* = 14.09) compared to White exonerees who served nearly four years less (*M* = 10.22). While a majority of the literature focuses on DNA exoneration cases, it is important to note that Non-DNA overturned convictions occur nearly five times as often, numbering 3153 (83.04%) compared to 644 exonerations containing DNA evidence (16.9%).

#### 3.1.2 Causes of wrongful conviction and their prevalence

The registry has categorized data based on a ‘well-known list of factors that are associated with exonerations, including eyewitness misidentification, false confession, perjury, false or misleading forensic evidence, and official misconduct [44]. The number of exonerations that each of these factors are currently associated with is displayed in Table 2.

**Table 2:** Exonerations by contributing factors in the USA.

Perjury/False Accusations	Official Misconduct	False/Misleading Forensic Evidence	Mistaken Identification	Inadequate Legal Defense	False Confessions
64.7% (N = 2456)	61.6% (N = 2306)	29.0% (N = 1100)	27.1% (N = 1030)	28.1% (N = 1066)	12.7% (N = 482)

Note: Totals for percentages exceed 100% due to multiple causes (about 80% of cases involved multiple contributing factors).

Notably, the two leading contributors to wrongful convictions in these data are perjury/false accusation and official misconduct. The importance of these factors, especially in comparison with other causes (e.g., false confession) appears to have surged during the last decade. We encourage much greater scrutiny of these two causes, especially by investigators and prosecutors who must address these egregious concerns in house, while keeping in mind they often do not occur in isolation.

#### 3.1.3 Exploratory analyses

One other interesting finding in relation to the US data (not available in relation to the England and Wales and Netherlands data) relates to when cases are associated with a single cause or multiple causes. As described by Toglia and Berman [8], the NRE exoneration cases differ on how many causes contributed to an innocent individual's wrongful conviction. This important difference has received little attention in the literature but is a critical distinction when conducting experiments and establishing reforms. In separating these exoneration cases, we denoted wrongful convictions based on a single cause as “pure”, while convictions involving multiple causes were labelled as “impure” [8,45]. This purity distinction turns out to be noteworthy for research that concerns unjust verdicts because an inspection of the NRE reveals that only about 20% of the cases are pure, while 80% are impure. This 80:20 ratio is not representative in the vast majority of experimental designs conducted in laboratory settings. This disproportionate ratio highlights the importance of employing archival methodologies to help understand and reform systemic flaws. While experiments examining factors contributing to wrongful convictions (e.g., mistaken identifications; false confessions) are crucial for developing theory and establishing clear cause and effect relationships, they are limited to strictly controlled environments encompassing a small number of manipulated variables. Additionally, unlike laboratory studies which simultaneously expose participants to the variables of interest, factors present in impure cases may arise at different stages of the investigative and trial process. Our analysis highlights the striking contrast between archival cases and experimental methodologies that should encourage researchers (as well as practitioners and policy makers) to consider this purity issue when examining wrongful convictions and establishing safeguards designed to prevent erroneous convictions. The degree of impurity can be easily computed, and it behooves researchers to account for differences between pure and impure contributing factors. When computing the number of overall contributing factors across all exoneration cases, the mean number of causes exceeds two per case (M = 2.20).

Broken down by race, the average number of causes were all near to the overall mean; the averages are 2.27, 2.19, and 2.10 for Blacks, Hispanics and Whites, respectively. Although the number of causal factors by race are similar, concerning differences were found for race when crossed with contributing factors. These racial shift patterns for the pre- and post-1989 eras are displayed in Table 3.

**Table 3:** Percentage of Wrongful Convictions by Race and Three Causes in the Pre-and Post-1989 eras.

	Race		MWID x Race		OM x Race		P/FA x Race	
	White	Minority	White	Minority	White	Minority	White	Minority
Era								
Pre-1989	52.2%	33.4%	50.2%	26.8%	58.2%	36.1%	52.2%	37.4%
Post-1989	33.1%	68.0%	17.2%	82.4%	26.6%	72.9%	29.5%	70.4%

Note: MWID (Mistaken Witness Identification); OM (Official Misconduct); P/FA (Perjury/False Accusations).

Identifying this shifting pattern for race is one of the benefits of exploratory analyses, which can highlight specific trends within the dataset. Attributing reasons for this data shift may provide insight into the impact of new policies or technological advancements. For example, we know this shifting pattern cannot be explained by the absence of racial discrimination in the Pre-1989 time period. In fact, that White prisoners were more likely to be exonerated prior to the availability of DNA testing speaks to discriminatory practices. This is because in Pre-DNA times, White individuals were relatively more favored due to “access.” Compared to minorities who were wrongly convicted, they were more likely to have their claims of innocence heard in a legal sense and had greater financial means to hire attorneys to fight their post-conviction cases. In the Post-DNA era, it was less about access and more about “opportunities” for all races to fight for justice. DNA evidence was a game changer for sure, however, two major events evened the playing field. These were the establishing of the Innocent Project in 1992 and the creation of Conviction Integrity Units (CIUs) in 2007 (these units are described in detail later in the article). With minorities comprising a majority of the prison population, it is understandable that a racial shift would reveal itself once reduction of disparities was supported by opportunity. Unfortunately, these troubling racial trends are not uncommon and extend to sentencing as well. When considering cases with the harshest penalties (Death, Life without Parole and Life), the same racial shift was found Pre-to Post-1989 (see Ref. [46,47]).

### 3.2 England and wales

The UK Miscarriages of Justice Registry contains data on convictions occurring from 1970 onwards in all of the United Kingdom's legal jurisdictions – England and Wales, Scotland, and Northern Ireland. Cases are eligible for inclusion in the registry if they involve a conviction that has been overturned on the basis of factual error (essentially amounting to factual doubts about the guilt of a defendant), and where the original conviction was not substituted for another conviction, the appellant was not re-tried and found guilty at a retrial and there is sufficient information about the case to allow inclusion (e.g., to verify that the case meets inclusion criteria; for more on inclusion criteria and registry compilation, see Helm, under review). At the time of writing, the registry contains 507 proven wrongful convictions – 420 in England and Wales, 50 in Northern Ireland, and 35 in Scotland. It is important to note that information available in relation to some cases was limited and, as a result, some data are missing from the registry (e.g., data on ethnicity are not available for all cases). An important note in relation to the England and Wales data, is that it is heavily influenced by the Post Office Scandal, a significant wrongful conviction that accounts for 97 cases in the current database (for more information on the Post Office Scandal, see [48]).

#### 3.2.1 Descriptive information

In the 420 cases in England and Wales, 82% of victims of wrongful convictions were male (and 16% were female). All offence categories are reasonably represented in the data. Twenty eight percent of cases involved a murder conviction, 16% involved a conviction for a sexual offence, 15% involved a conviction for robbery or burglary, 12% involved a conviction for theft or fraud, 10% involved a conviction for false accounting, 9% involved a conviction for manslaughter or a non-fatal offence against the person, 4% involved a conviction for a drugs offence, and 14% involved another conviction. The average time spent incarcerated among those included in the registry who did spend time in prison (and whose time in prison was known) was 4.83 years (the SD was not provided, thus not reported here), with a combined total of 2028.5 years wrongly served in prison.

In those cases, approximately 75% involved a defendant classified as ‘White,’ approximately 13% involved a defendant classified as ‘Black’ and approximately 11% involved a defendant classified as Asian. These data should be treated with caution due to the large amount of missing data relating to ethnicity, as well as potential difficulty in accurately ascertaining ethnicity and therefore potential for error in underlying sources. However, broadly, the data suggest an overrepresentation of those of Black ethnicity in recognised wrongful convictions compared to their representation in the general population at 2.5% [49], consistent with overrepresentation elsewhere in the criminal justice process (see e.g., Ref. [50]). Case numbers are currently too small for a reliable comparison of time served by ethnicity. While the registry does not currently code cases according to whether convictions were overturned based on DNA evidence, a review of the data suggests that relatively few cases were overturned on this basis. However, recently DNA evidence has been influential in overturning wrongful convictions, as in the case of Andy Malkinson (conviction overturned in 2023; [51]), the case of Peter Sullivan (conviction overturned in 2025; [52]), and the case of Victor Nealon (conviction overturned in 2013; [53]).

#### 3.2.2 Causes of wrongful conviction and their prevalence

Eighty-seven cases involved a defendant who initially pleaded guilty (although 77 of these cases were associated with the Post Office Scandal; for a discussion of guilty pleas in the registry data, see Ref. [35]). Six key factors associated with wrongful convictions were identified in a review of the data – inadequate disclosure, official misconduct, witness evidence from a non-complainant, confession evidence, false or misleading forensic evidence, and witness testimony from a complainant. The registry does not categorize cases based on whether they have a single cause or multiple causes. The number of cases that each of the factors are currently associated with (including and excluding Post Office data) is displayed in Table 4.

**Table 4:** Exonerations by contributing factors in England and wales.

Inadequate Disclosure	Official Misconduct	Witness Evidence from a Non-Complainant	Confession Evidence	False or Misleading Forensic Evidence	Witness Testimony from a Complainant
35% (N = 149) [18%]	28% (N = 117) [36%]	24% (N = 102) [31%]	18% (N = 76) [22%]	16% (N = 66) [20%]	12% (N = 52) [31%]

Note: Percentages in square brackets are the percentages excluding Post Office Data. Totals for percentages exceed 100% due to multiple causes.

It is important to note that some subjective judgment was involved in forming these categorisations. For example, the decision was made to split misleading witness evidence based on whether the evidence came from a non-complainant or a complainant rather than by memory error or dishonesty, due to difficulty in making reliable judgments about the reasons that a witness gave an apparently misleading account in some cases. Interestingly, other research, which has sorted registry data to examine cases involving identification evidence specifically, suggests that there may be fewer recognised wrongful convictions resulting from inaccurate eyewitness identifications in England and Wales than in many other jurisdictions (see Ref. [14,23], Chapter 3).

#### 3.2.3 Exploratory analyses

Two hundred and sixty convictions in the database cases were quashed following a referral from the CCRC, representing the majority of cases in the database, despite the CCRC only having begun operations in the 1990s. The registry also includes data on majority verdicts. Although information on whether a verdict was unanimous or majority is not available for many cases, the registry includes at least 46 cases in which the conviction occurred on the basis of a (super) majority, rather than unanimous, jury verdict. These cases have been analysed in work linking majority verdicts to wrongful conviction (see Ref. [54]).

### 3.3 The Netherlands

The EUREX database has currently documented 144 exoneration cases across 20 European countries, arising since 1970. The registry defines exoneration as the post-conviction re-examination of a case resulting in a final judgement that overturns a prior conviction. Specifically, an exoneration according to the EUREX definition may take one of four forms: acquittal of all charges by an authorised government official or agency; a declaration of factual innocence by an authorised government official or agency; dismissal of all charges by a court or prosecutor; or a complete pardon by a governor or other competent authority. This rather conservative definition is crucial, given the diversity of legal systems across Europe. European jurisdictions differ substantially in their procedural frameworks, encompassing both inquisitorial and adversarial traditions, and vary in the mechanisms through which convictions may be overturned, including retrials, appeals, pardons, and acquittals. In the absence of a harmonised legal concept of exoneration across these systems, the same underlying phenomenon may be labelled and processed differently from one country to the next. Furthermore, data are collected through publicly available information, as access to court files remains restricted in many European countries. As a result, the picture painted by EUREX is likely incomplete. Germany contributes the largest number of cases (n = 33), followed by Italy (n = 18), the Netherlands (n = 15), Sweden (n = 14), Spain (n = 11), France (n = 10), and Norway (n = 9). The remaining cases are distributed across Ireland, Ukraine, Iceland, Romania, Switzerland, Austria, Hungary, Finland, Poland, Denmark, Greece, Czechia, and Bulgaria. Ethnicity data are not recorded in EUREX, reflecting the legal constraints on ethnic data collection across European criminal justice systems, in contrast to registries such as the National Registry of Exonerations in the United States. As explained above, the analysis in this article will focus specifically on the Netherlands.

#### 3.3.1 Descriptive information

Fifteen Dutch cases are recorded in the EUREX dataset. As in the broader European sample, exonerees were predominantly male (n = 13, 86.7%), with two (13.3%) female exonerees. As an aside, readers may notice that the percentage of female cases is quite similar (low) across the three registries; and of interest is that the crimes for which women are wrongly convicted share similarities as well. In all registries, aside from homicides, a significant proportion of females were falsely accused of crimes involving child and caretaker harm (e.g., harm to infants and/or patients). In line with the general pattern of the EUREX data, wrongful convictions in the Dutch cases were heavily concentrated in the category of (attempted) murder or manslaughter (n = 11; 73.3%). The remaining cases involved other offenses (n = 2, 13.3%), a violent offence (n = 1; 6.7%), and arson (n = 1; 6.7%). No sexual offence cases were recorded in the Dutch subsample. Notably, only 2 of the 15 Dutch cases involved DNA as an exonerating factor, reflecting a broader European pattern in which DNA-based exonerations remain rare compared to the United States.

#### 3.3.2 Causes of wrongful convictions and their prevalence

The factors identified as being causes of wrongful conviction in Dutch cases, and more generally exonerations documented in EUREX, include false confessions, false or misleading forensic evidence, perjury or false accusation flawed eyewitness identification procedures, and other factors. The number of cases that involve each of the factors is displayed in Table 5.

**Table 5:** Exonerations by contributing factors in the Netherlands.

False Confessions	False or Misleading Forensic Evidence	Perjury or False Accusation	Other Factors	Flawed Eyewitness Identification Procedures
47% (N = 7)	33% (N = 5)	27% (N = 4)	13% (N = 2)	7% (N = 1)

Note: Totals for percentages exceed 100% due to multiple causes.

#### 3.3.3 Exploratory analyses

Of the 15 Dutch cases, 12 (80%) involved a crime which occurred but was not committed by the exoneree. In one case, no crime occurred, in another, no crime could be proven, and yet another had no available information regarding this. A case review unit was involved in 4 of the 15 (27%) Dutch cases. Notably, official misconduct did not appear as a contributing factor in any Dutch case, which will be discussed further below.

## 4 Key findings, similarities and differences

The comprehensive analysis of wrongful conviction databases across the United States, England and Wales, and the Netherlands illuminates both shared patterns and distinct nuances in the landscape of wrongful convictions. The data are clear! Wrongful convictions are a global phenomenon and not the product of one system or culture. Similarly, the data (regardless of registry or country) demonstrate that wrongful convictions are not the result of isolated errors remedied with an easy solution. Instead, they constitute a complex intersectional phenomenon that requires a more nuanced approach for both understanding and reform.

Collectively, the data repositories underscore the multifaceted and deeply entrenched nature of wrongful convictions, revealing patterns of racial disparity, systemic failures and the necessity for comprehensive reforms. The co-occurrence of causal factors and the purity distinction (between single- and multiple-cause cases) drawn explicitly in relation to the US data emerges as a factor that should be assessed in analyses of data in other jurisdictions, and a consideration for future studies which can better inform policymakers and respective reforms. Potential differences based on ethnicity also warrant further research, particularly in England and Wales where they are relatively understudied, partly due to a lack of available data.

There are also some interesting differences between jurisdictions, although these are difficult to interpret reliably. One interesting difference is the absence of wrongful convictions associated with official misconduct in the Netherlands. This contrasts with the European-wide picture where it was present in 17.4% of cases, as well as with the US (as the second most common cause) and England and Wales data. It may represent a less significant role of official misconduct in the Netherlands, a product of the number of cases, or simply, less reporting of misconduct in the publicly available sources which EUREX relies on for case information. Another interesting difference is the higher proportion of cases involving guilty pleas in the USA compared to England and Wales, which may reflect the more extreme incentives that defendants face to plead guilty in the USA and may reflect the difficulty appealing a conviction following a guilty plea in England and Wales (although appealing following a guilty plea can also be challenging in the USA, as noted earlier).

However, some similar themes also emerge among the three jurisdictions, despite their differences. These include important roles of false confession, evidence from eyewitnesses (including perjured evidence and mistaken evidence), and false or misleading forensic evidence in wrongful conviction cases. It is also noteworthy that DNA is still only utilized in a minority of cases, even in the USA where it appears to be utilized far more frequently than in England and Wales and the Netherlands. The presence of these similarities suggests that proposed and actual reform in one jurisdiction may be informative in the others. We now move to considering some recent reforms targeted at addressing wrongful convictions, and where reform in one jurisdiction might usefully serve as a blueprint to be considered by others. The reviews considered here are intended to be relevant examples, rather than a comprehensive overview of potentially relevant reforms.

## 5 Reforms to address wrongful convictions

### 5.1 The utilization of DNA evidence

Recognizing the value of DNA testing, all 50 states in the USA now have statutes that allow for post-conviction DNA testing upon a motion being filed with the trial court. The rub here is that the standard for review varies by state for the motion to be granted [55]. Some states, like Oregon, are more encompassing and allow for DNA testing at any time and by a showing that there is a reasonable probability that the DNA testing would have prevented the petitioner's conviction, while other states, like Delaware, have strict deadlines and require proof that the DNA would prove the innocence of the petitioner, and yet other states, like Florida, will not allow post-conviction DNA testing for individuals who entered a plea to resolve the case. Of course, post-conviction DNA testing is only possible if the evidence was stored and is available for testing, and even this can be an issue depending upon the state and its laws for collection and preservation of evidence [56,57]. However, this approach to formalizing the right to post-conviction DNA testing may helpfully be drawn upon in other jurisdictions. This issue is particularly topical in England and Wales, where calls for greater testing have intensified following the recognition of the high-profile wrongful conviction of Andy Malkinson, whose conviction was overturned based on DNA testing (see Ref. [58]: https://www.theguardian.com/law/2024/apr/15/dozens-of-jailed-people-may-get-new-dna-testing-after-andrew-malkinson-exoneration).

It is important to reemphasize that as useful as biological evidence is in the DNA era (Post-1989), for most crimes DNA is unavailable and thus not a factor. The public has often heard about “DNA exonerations,” However, actually most wrongful convictions are overturned on bases other than the presence of DNA. As reported earlier, only 1 in 6 cases in the NRE are DNA exonerations.

### 5.2 The treatment of eyewitness identification evidence

One issue identified as underlying wrongful convictions in each jurisdiction is issues with eyewitness identification evidence. This reality is perhaps unsurprising as this type of evidence is widely utilized and, yet, susceptible to error. The issue of mistaken witness identification has spawned thousands of cognitive and social psychology experiments over the last 40 years (see Ref. [59]), providing insight into, among other things, memory errors in lineup and show-up decisions, comparisons of simultaneous vs. sequential lineups, confidence inflation of faulty identifications, fair vs. biased lineups and choosing tendencies in suspect-present vs. suspect absent lineups [59,60]. This work has been translated into legal procedures and/or recommendations designed to protect against wrongful conviction (see [61] regarding “six claims on eyewitness identification” and the White Papers, [62,63]), but identification evidence seems to persist as a cause of wrongful convictions, consistent with historical accounts [9]. A recent case involving identification evidence in the NRE, for example, involved a conviction in 2023; that of Ahmad Gatlin. This problem may result from a continuing lack of education relating to the reliability of memory and factors underscoring its reliability and unreliability (for discussion in England and Wales in the context of the CCRC, see Ref. [64]).

In the United States, an interesting case demonstrates a potential lack of knowledge in this area. In 2016, the United States Court of Appeals for the Third Circuit sat on *Dennis v. Secretary, Pennsylvania Department of Corrections,* 834 F.3d 263 (3rd Cir. 2016). In that case, the prosecution primarily relied on the testimony of three eyewitnesses to convict Dennis of murder. Dennis maintained from the beginning that he was not involved in the crime and provided compelling alibi evidence that was discounted by the prosecution. One alibi witness was Dennis’ father, one was a woman on the city bus (who thought she had seen Dennis at a time other than Dennis had said), and the other witnesses were friends from a singing group where Dennis said he was participating in a rehearsal. Specifically, Dennis claimed that he was on a bus traveling to singing rehearsal at the time the crime was taking place. Dennis claimed to have seen a woman he knew while on the city bus. This woman acknowledged seeing Dennis that day but believed it was later in the day than Dennis had claimed. Post-trial, it was discovered that the prosecution had withheld a number of documents, including a receipt that corroborated the timeframe provided by Dennis for seeing the woman on the bus. The case was reversed because the prosecution had failed to disclose the documents to the defense; in the United States this omission is referred to as a Brady violation (*Brady v. Maryland,* 373 U S. 83, 1963).

A fascinating aspect of the case was the disparity of opinions held by the judges regarding the credibility of eyewitness identification testimony. Some of the judges on the panel recognised the fallibility of eyewitness identification and held there was a reasonable probability that the outcome of the trial would have been different had Dennis had the documents corroborating his alibi defense, while the dissenting judges placed great reliance on the eyewitnesses' identification of Dennis, noting “the evidence against Dennis was strong—it is hard to discount the identification testimony of three eyewitnesses.” *Id*. at 357. This case led the Third Circuit to create a first-of-its-kind Task Force on Eyewitness Identification with the goal of educating those in the criminal legal system on the issues that “surround eyewitness identifications and to minimize their role in erroneous convictions” [65]. The Task Force was comprised of 18 members, which included federal judges from each district within the Third Circuit, prosecutors, defense attorneys, law enforcement officials, and researchers. The group convened for three years, ultimately authoring a final report in 2019 to serve as a practical guide and tool for practitioners [65]. Like other academic bodies around the country, the Task Force recommended science-based protocols for law enforcement's administration of photospreads and eyewitness identification in criminal cases, recognizing that the memory of an eyewitness should be treated with the same care as the physical crime scene itself [66]. While many of the proposals made by the Task Force mirror recommendations already in place in other jurisdictions (e.g., some recommendations, such as collecting immediate confidence statements when an initial identification is made, are relatively novel, and could helpfully be incorporated into practice in other jurisdictions [61,62]).

Of course, beyond identification frailties, eyewitnesses can not only be mistaken in their testimony about the events concerning a crime, but may also be deliberately deceptive, a form of perjury. As noted earlier, distinguishing between these possibilities is often difficult, and many registries (including the UK Miscarriages of Justice Registry and EUREX) do not make this distinction (but see e.g., Gross & Shafer, 2012). Similarly, in the NRE for the United States, this distinction is unclear as the Perjury/False Accusation (P/FA) was not designed to differentiate between mistaken testimony and deliberate deception. Thus, types of false testimony are not separated in the systems examined in this paper. Reforms aimed at reducing eyewitness error are unlikely to address instances of intentional deception, and few reforms to date have specifically targeted this issue.

### 5.3 Forensic science evidence

Another important factor that continues to be associated with wrongful convictions across jurisdictions is forensic science evidence. In the USA, many states have established forensic science oversight bodies, which vary in form (i.e., structured as commissions or boards) as well as the myriad roles and responsibilities associated with these bodies [67]. In all cases, however, these forensic science oversight entities are designed to help ensure complete, accurate, and, importantly, timely evidence collection. They also oversee forensic analysis and the transparent, efficient, and effective operation of publicly funded crime laboratories. At the states-level, these official groups coordinate with federal standards (e.g., the American National Standards Institute) and accreditation panels (e.g., the American Academy of Forensic Sciences). Momentarily, we will address, in a global fashion, forensic science errors as they relate to wrongful convictions.

Regarding England and Wales, weaknesses in forensic science evidence have recently been considered in depth as part of two reviews ([68]; 2026; [69]). A number of recommendations have come out of these reviews, that have the potential to improve the treatment of forensic science evidence in England and Wales and which could, potentially, be informative more broadly. One recommendation, which has been substantially implemented, was giving more concrete powers to the Forensic Science Regulator, the person responsible for standards and compliance in forensic science. The regulator now has clear statutory powers to set quality standards for forensic science activities and to investigate suspected non-compliance (for discussion of these changes, see Ref. [70]). In a similar vein, we note the importance of strengthening the independence of forensic science investigation from policing and the need for more research funding in forensic science. This includes additional support for work in digital forensics, which has been identified as potentially being an important modern contributor to wrongful convictions [35,69].

The Netherlands employ a distributed style of forensic science oversight, centered around the *Netherlands Register of Court Experts* (NRGD), which operates as the main national entity for regulating forensic practices. The NRGD establishes competency standards, accredits forensic experts, and retains a public inventory to facilitate the appointment of qualified specialists in criminal proceedings. Laboratory-level quality assurance is achieved and maintained through mandatory accreditation, supervised by the *Dutch Accreditation Council* (Raad voor Accreditatie), which conducts frequent external audits of forensic laboratories.

All three jurisdictions highlight the importance of forensic science. The use of forensic evidence (including collection, preservation, and testing) by criminal justice systems is crucial evidence for arrests and the prosecution of defendants. Rightful convictions are often decided based on the accuracy and relevance of such evidence. However, in each of the wrongful conviction registries we examined, the opposite effect was found showing the hidden dangers of flawed forensic science methods. For example, False or Misleading Forensic Evidence (F/MFE) occurs with substantial frequency as displayed in Table 2, Table 4, Table 5. Edmond and Vuille [71] explored weaknesses in forensic science evidence in the USA, Australia and Switzerland. Similar to our multiple database comparisons, the latter criminal justice system is inquisitorial, while the other two countries adhere to an adversarial legal framework. Based on their investigation, Edmond and Vuille [71] described “how three different criminal justice systems each have failed to identify or credibly respond to deep structural and endemic problems with many types of forensic science and medicine evidence routinely used by investigators and prosecutors.” (p.221). Although isolating one particular contributing factor to wrongful convictions is not our primary focus, we appreciate Edmund and Vuille's concrete suggestions to heighten awareness and improve the use of valid forensic science methods and technologies, especially as these can only aid in reducing wrongful convictions.

Earlier, we drew attention to wrongful conviction cases involving women. Therein, we noted across jurisdictions the similarity in low frequency of such cases, as well as parallels in the crimes for which females have been exonerated. Having now highlighted forensic errors, we further explored if these mistakes were disproportionate when considering the gender of the exoneree. Specifically, are women's cases more likely to be influenced by such errors? We examined this by delving deeper into the data for the False or Misleading Forensic Evidence (F/MFE) contributing factor. In the NRE, F/MFE influenced wrongful convictions in over 1100 cases, that impacted 12% of women and 88% of men. To contextualize these figures, on a proportional basis, 40.3% of women's unjust convictions involved false or misleading forensic information, while the comparable percentage for men was only 28.1%. In addition, this cause was a pure contributing factor in 10.9% of women's F/MFE cases, while for men the corresponding purity percentage was only 4.3%. These two contextualizing sets of figures support the argument that in the USA women are disadvantaged by flawed forensic science evidence.

In England and Wales, 420 total wrongful convictions are documented in the Miscarriages of Justice Registry; broken down by gender, 67 (16%) cases involved women exonerees, compared to 353 (84%) cases for men. Proportionately, 18 (26.9%) of the 67 cases included F/FME as a contributing factor for women, while 48 (13.6%) of the 353 cases involved the F/FME cause for men. This pattern mirrors the findings from the USA showing that in England and Wales, women are also disproportionately disadvantaged by unreliable forensic science evidence. Due to a small sample size (4 men; 1 woman) comparisons for sex involving F/FME as a contributing factor in the Netherlands were not computed. Thus, meaningful conclusions relating F/FME to sex of exoneree are clearly premature for Dutch wrongful convictions.

## 6 Organizations assisting with the identification of wrongful conviction

### 6.1 Criminal justice institutions

In the USA, one innovation with the potential to be helpful in the identification of wrongful convictions has come from prosecutorial offices themselves – the creation of Conviction Integrity Units (CIUs). CIUs are specialized divisions within the prosecutor's office that review claims of factual innocence, made by those who were convicted of a crime within that jurisdiction [72]. The creation of CIUs within prosecutorial offices is not without professional risk, as it invites scrutiny from within and outside the office, but has become a consequential tool for justice reform. It is generally accepted that the first CIU was created in 2007 by the newly elected prosecutor in Dallas County, TX. At that time, Dallas County already had the most DNA exonerations of any county across the country. The purpose of the CIU was to review cases where DNA existed and would have been dispositive but had not been tested. To date, this CIU has been responsible for approximately 32 exonerations [17]. The creation of this unit changed the dialogue nationwide around the role of the prosecutor's office in addressing wrongful convictions and fostered a philosophical shift from winning cases at all costs to pursuing justice. In 2007 there was only one CIU, whereas there are now approximately 110 CIUs around the country, with additional units continuing to be established [17]. As CIUs become more commonplace, so too has recognition that mistakes must be rectified when identified. Typically, a CIU is run by an attorney with prior criminal defense experience, and, because the work of a CIU is extra-judicial, the CIU can reinvestigate a case without being constrained by post-conviction procedural bars and time limits typically encountered by a defendant or their counsel during the post-conviction litigation or appellate process. A CIU investigation can include document review, witness interviews, forensic testing and analysis, expert consultation, crime scene recreation, and any number of investigative techniques. Over the past decade, CIUs have been responsible for more than 600 exonerations [17]. In addition to the work of innocence organizations, CIUs have been a significant driver of exonerations nationwide.

The CCRC in England and Wales was designed to provide a fresh, independent, and ultimately more effective approach to the investigation of wrongful convictions (taking over a role that had previously been held by the Home Secretary). In practice it has been the subject of a reasonable amount of criticism for failing to identify now proven wrongful convictions (e.g., the cases of Andy Malkinson, Victor Nealon, Oliver Campbell, and Peter Sullivan; see Ref. [[51], [52], [53],^73^]) and having (partly as a result of the “real possibility” test) too much deference to the Court of Appeal (see, for e.g., Ref. [64,74]). However, it has, ultimately, been responsible for the identification of a significant number of wrongful convictions in England and Wales, and organizations based on the CCRC model have been created (and utilized successfully) in other jurisdictions (see Ref. [23], Chapter 5). The role of the CCRC, alongside the rules on appeals more broadly, is currently being considered by the Law Commission of England and Wales, an independent body charged with keeping the law of England and Wales under review and recommending reform where necessary [38].

In the Netherlands, a sharp turning point occurred in 2004 when the wrongful conviction in the Schiedam Park murder case was overturned. The case involved coercive interrogations followed by a false confession, which led to tunnel vision in the police investigation and convictions by all courts. The innocent suspect spent four years in prison, until the real perpetrator confessed and his DNA was found to match the DNA recovered at the crime scene. This case prompted major reforms in the Dutch criminal justice system, such as the introduction of a ‘devil's advocate’ approach in all serious police investigations and a move towards less accusatorial and more information-gathering investigative interviewing approaches [75]. It was also what led to the establishment of the Committee for the Review of Closed Criminal Cases (CEAS) in 2005 (later replaced by the ACAS), with the mission to evaluate closed criminal cases and advise on whether renewed investigation or revision is warranted.

#### 6.1.1 Not for profit organizations

Not-for-profit Organizations have been extremely important in the recognition of wrongful convictions. The Innocence Project of New York, created in 1992, has been responsible for over 250 exonerations using DNA that was not tested prior to conviction. Out of that success grew a movement in the USA leading to more than 60 independent innocence organizations that comprise the “Innocence Network.” These Innocence Network organizations are located in every state across the country and are collectively responsible for approximately 850 exonerations [17]. However, in England and Wales, they have faced challenges. The UK Innocence Network, which at one time included 26 university innocence projects and one corporate law firm project, closed operations in 2014 (see Ref. [23], Chapter 5 for discussion relating to this network and its closure). Pro bono support for those who allege that they have been victims of wrongful convictions is now only provided by a relatively small number of organizations, including APPEAL, Inside Justice, and the Cardiff University Innocence Project.

In the Netherlands, the Schiedam Park Murder case in 2000 led not only to significant criminal justice reforms but also marked the starting point of an influential not-for-profit justice initiative known as Project Reasonable Doubt (http://projectgeredetwijfel.nl/). This project involves groups of students, supervised by project staff, who investigate closed criminal cases in which doubts arose regarding the conviction. This entails an extensive analysis of the case dossier in combination with studying scientific literature and consulting experts on forensically relevant topics that played a role in the conviction, ranging from estimating the time of death to the psychological state of the perpetrator. Since its inception in 2002, close to 500 students have contributed to Project Reasonable Doubt, investigating some 60 cases, resulting in 27 publications [76]. Three cases were eventually re-opened and in two out of three, the originally convicted person was ultimately acquitted.

Project Reasonable Doubt investigations often involve the design of scientific experiments to answer questions that are so specific to the case that they cannot be answered based on general scientific insights. For example, in one case, a neighbor was convicted of murdering a woman and setting her house on fire, largely because his DNA was found in material taken from beneath her fingernail [77]. He argued that his DNA must have gotten underneath her fingernails during an argument two weeks earlier, when she had scratched him so hard that her gel nail broke, which she repaired herself. Experts considered it unlikely that his DNA remained under her nail for that long, survived a fire, and endured exposure to firefighting foam, especially given that no other DNA was detected, not even that of her infant daughter. However, Project Reasonable Doubt researchers formulated and tested a novel explanation: that his DNA had become encapsulated *inside* the gel nail during the repair. Chemical experts confirmed that the type of gel used would not necessarily degrade DNA. The team then tested the scenario: four students had donor blood placed under their nails before receiving gel nail treatments. When the gel nails were removed two weeks later, donor DNA was detected in six of the eight samples. This provided strong new evidence that the alternative explanation was plausible and warranted serious consideration. Other examples of case-specific Project Reasonable Doubt experiments have included tests of whether blood could be detected in a washing machine after washing bloody clothes (it could; Van Dobben et al., 2018) and whether a dead body of a certain size and weight could be lifted over a bunk bed (it could not). These experiments provide valuable new insights that cannot be obtained by consulting scientific literature or experts, and the model may helpfully be utilized in other jurisdictions.

## 7 Conclusions

The investigation into multiple international data sets illustrates the prevalence of wrongful convictions and suggests they may be underestimated. Although this ubiquitous phenomenon has become more visible in the past 20 years, the results of our analyses provide an important step in diagnosing global systemic failures. Alarmingly, the injustices surrounding wrongful convictions emerge regardless of system (e.g., adversarial vs. inquisitorial) or geographical location. Analyses using global databases highlight the importance of examining wrongful convictions and reform efforts from an international viewpoint. This holistic approach allows varying jurisdictions to learn from each other to ultimately advance the efforts of researchers, policy makers, and criminal justice practitioners in transforming their systems. In essence, this is a point recently highlighted by Roach [78] wherein he noted that it has not gone unnoticed that each of the systems we have discussed can inform the other regarding the reduction of errant convictions. As examples, Roach explains that the inquisitorial approach involves far less offering of plea deals and a strong focus on seeking the truth, both worthy of greater consideration by countries who have adopted an adversarial system. On the other hand, he indicates that significant transparency in fact-finding and the strength of challenging a witness's testimony via the process of cross examination in the adversarial approach, merits further attention of jurisdictions with inquisitorial systems. Playing to each other's strength, could be framed with the name that Roach [78] titles a section in chapter 4: “Is the Grass Greener on the Other Side of the Adversarial/Inquisitorial Divide?” Maybe. Yet this chapter echoes a conclusion of the present article: regardless of the criminal justice system, wrongful convictions happen.

Several noteworthy insights emerged from our results. First, wrongful convictions are not simply the result of one contributing factor or integral to any one system. Instead, erroneous convictions are a culmination of a myriad of interactive factors embedded within a system, administered and enforced by people who are prone to the same structural prejudices as the rest of society. Second, these findings, in conjunction with existing literature, serve as a foundation for formulating future policies and reforms. They provide crucial, evidence-based strategies to inform and refine subsequent policy development and reforms. For example, each database uniquely contributes to form a useful blueprint to assist others in developing their own database tailored to their respective laws, legal system and jurisdiction.

The present findings also provide insights for current and future database managers regarding the coding of case variables and the types of analyses needed to understand the contributing factors and trends, which are essential for developing effective reforms. Furthermore, this holistic perspective contributes to a new understanding of how archival databases can be utilized to inform experimentation. This approach can be viewed prescriptively, as captured by Toglia and Berman [8], with the strategy “looking back to move forward”. That is, looking back archivally to move ahead with new, creative experiments. The authors went a step further by proposing that experimental findings could prompt renewed examination of archival material, thereby generating an ongoing cycle of reflection and experimentation. Finally, it is important to note that the data and conclusions drawn from global databases are only as robust as the information they contain. Given the lack of standardization and the absence of an overarching international legal framework governing a defendant's right to innocence, the sum of combined databases may present a different gestalt than any single source alone. This underscores the importance of data completeness in obtaining an accurate understanding. We emphasize this point since missing data persists as a reoccurring obstacle in mining archives. To maximize the benefits of using global databases to inform policy and reforms, it behooves criminal justice practitioners regardless of system or geography to establish and maintain databases in terms of contemporariness, accuracy, and extensiveness.

## 8 Future directions and final remarks

Although the examination of these databases portrayed many differences, importantly, they also displayed numerous similarities. While differences were obviously anticipated, the similarities across databases were striking. The similarities in factors associated with wrongful convictions across databases means that, despite procedural differences, jurisdictions may be able to learn from each other in the development of practices to target wrongful convictions and thus may be acted upon using similar reforms. Similarly, the differences found in our investigation can be helpful in moving towards what Garrett et al. [79] labelled as an international innocence gap or the right to recognize the accused entitlement to claim innocence internationally. Through a global lens, the combination of the likenesses and variances found in our analyses demonstrate a step in the right direction in moving towards international legal and policy reforms for claims of innocence addressing the universal problem of wrongful conviction.

## Declaration of generative AI use

During the preparation of this manuscript, the authors used Claude (Anthropic) and Microsoft Copilot to reformulate sentences and improve the precision and clarity of the language in parts of the manuscript, and to assist with formatting one of the tables. After using these tools, the authors reviewed and edited the content as needed and take full responsibility for the content of the publication.

## CRediT authorship contribution statement

**Michael P. Toglia:** Conceptualization, Project administration, Supervision, Writing – original draft. **Rebecca K. Helm:** Funding acquisition, Writing – original draft. **Garrett L. Berman:** Writing – review & editing. **Shelley L. Thibodeau:** Writing – review & editing. **Annelies Vredeveldt:** Writing – review & editing. **Jennifer M. Schell-Leugers:** Writing – review & editing.

## Declaration of competing interest

The authors declare that they have no known competing financial interests or personal relationships that could have appeared to influence the work reported in this paper.
